# Pneumococcal Myocarditis in an Immunocompetent Young Adult: A Case Report and Literature Review

**DOI:** 10.7759/cureus.92959

**Published:** 2025-09-22

**Authors:** Khalil Tritar, Souha Hannachi, Rym Abid, Riadh Battikh

**Affiliations:** 1 Infectious Disease, Hôpital Militaire Principal d’Instruction de Tunis, Tunis, TUN

**Keywords:** bacteriemia, cardiac magnetic resonance imaging, cmri, community-acquired pneumonia (cap), pneumococcal myocarditis, streptococcus pneumoniae

## Abstract

Pneumococcal myocarditis is an uncommon complication of invasive pneumococcal disease, although experimental data suggest that cardiac involvement may be more frequent than clinically recognized. We describe the case of an 18-year-old immunocompetent male who developed community-acquired pneumonia complicated by myopericarditis. *Streptococcus pneumoniae* was isolated from blood cultures. Transthoracic echocardiography revealed a reduced left ventricular ejection fraction (40-45%) with pericardial effusion, and cardiac MRI confirmed functional impairment without evidence of necrosis or inflammation. The patient was treated with a 14-day course of intravenous antibiotics alongside supportive care, resulting in full clinical and cardiac recovery. This case emphasizes the under-recognized risk of myocarditis in the context of pneumococcal pneumonia, underscores the importance of routine cardiac evaluation, and highlights the need for heightened clinical awareness to ensure timely diagnosis and management.

## Introduction

Invasive pneumococcal disease (IPD) remains a major cause of morbidity and mortality worldwide, particularly in young children, elderly patients, and individuals with chronic comorbidities. *Streptococcus pneumoniae* is the most common cause of community-acquired pneumonia (CAP) and is associated with high rates of bacteremia and cardiovascular complications among hospitalized adults [1]. In addition to pneumonia, meningitis, and sepsis, pneumococcal infection can also affect the cardiovascular system through both direct and indirect mechanisms [2].

Experimental studies have demonstrated that pneumococci can translocate into the myocardium, forming microlesions and inducing cardiomyocyte necrosis [3-5]. Clinical observations further suggest that cardiac complications during pneumococcal infections, including arrhythmias, pericarditis, and myocardial dysfunction, may be under-recognized [6-8]. Myocarditis, in particular, has only rarely been reported, despite the established pathogenic potential of the organism [9-12].

We present here the case of an immunocompetent young adult with pneumococcal pneumonia complicated by myopericarditis, followed by a review of the available literature.

## Case presentation

An 18-year-old immunocompetent male presented to the emergency department on the third day of a febrile illness with chills and malaise. Two days prior, he had developed right-sided chest pain with productive cough and greenish sputum. On admission, he was febrile (41°C), tachycardic (105 bpm), hypotensive (100/70 mmHg), and mildly tachypneic (20 breaths/min). Auscultation revealed right-sided crackles and a pericardial friction rub.

The electrocardiogram showed a regular sinus rhythm without conduction or repolarization abnormalities.

Laboratory tests revealed leukocytosis (28 × 10⁹/L) with neutrophilic predominance (26.8 × 10⁹/L), elevated C-reactive protein (249 mg/L), and high-sensitivity troponin (272 ng/L). Other laboratory results are summarized in Table 1.

**Table 1:** Patient laboratory results and reference values CRP: C-reactive protein, LDH: lactate dehydrogenase, CPK: creatine phosphokinase

Laboratory Parameter	Patient Value	Reference Range
Leukocytes	28 × 10⁹/L	4-10 × 10⁹/L
Neutrophils	26.8 × 10⁹/L	1.8-7 × 10⁹/L
CRP	249 mg/L	<5 mg/L
Procalcitonin	19 ng/L	<0.5 ng/L
Prothrombin time	38%	70-100%
LDH	434 U/L	190-390 U/L
CPK	638 U/L	15-200 U/L
High-sensitivity troponin (day 1)	272 ng/L	<19 ng/L
High-sensitivity troponin (day 2)	210 ng/L	<19 ng/L

**Figure 1:**
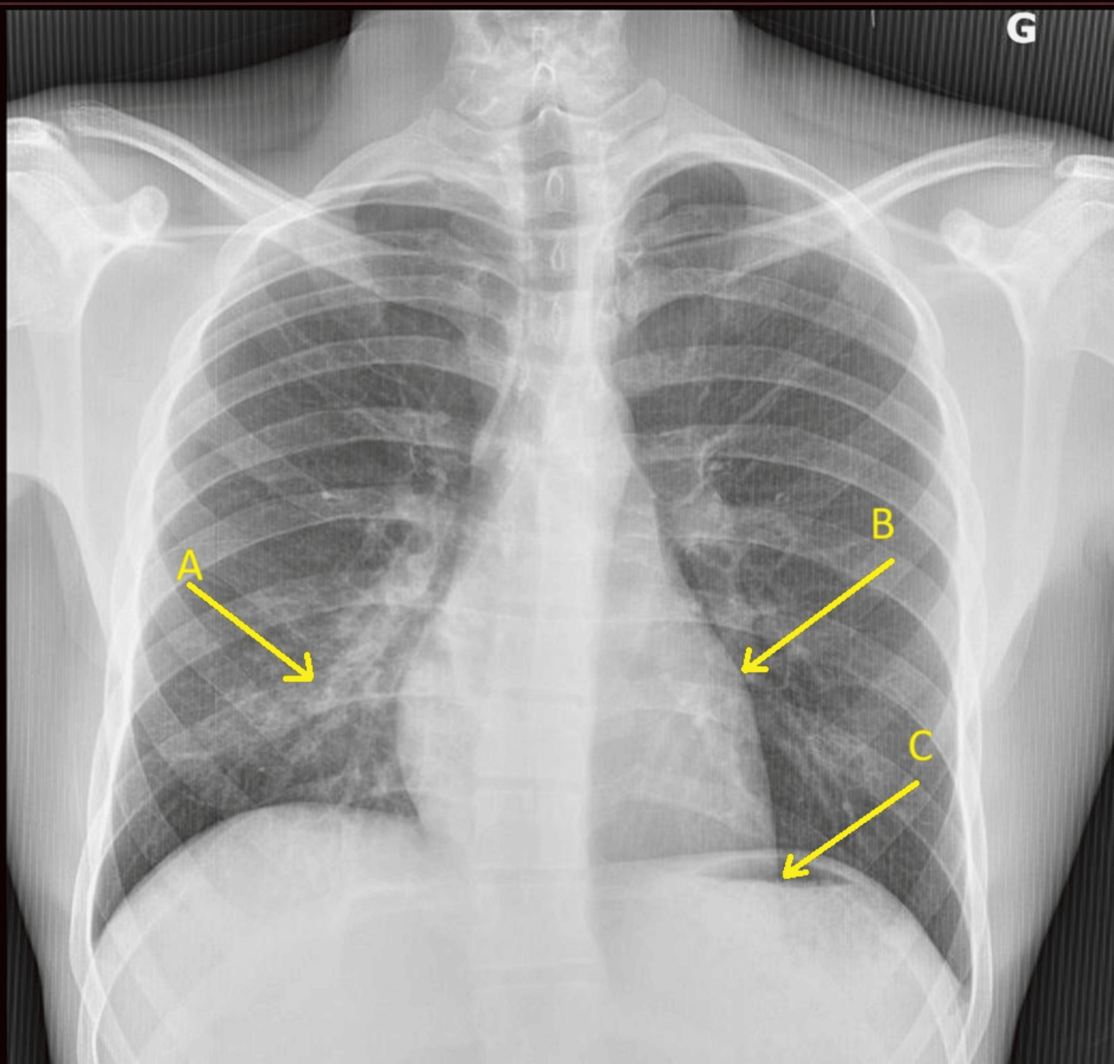
Chest X-ray on admission Chest X-ray showing right basal reticulonodular opacities (A) and cardiac silhouette abnormalities, including a bulging left middle arch (B) and a subdiaphragmatic apex (C).

Chest X-ray revealed right basal reticulonodular opacities and an abnormal cardiac silhouette with left heart border bulging and a subdiaphragmatic apex (Figure 1).

The patient was hospitalized and started on empiric intravenous amoxicillin‑clavulanic acid (1 g/200 mg, three times daily) for CAP. Intravenous gentamicin (5 mg/kg/day) was also administered early due to initial suspicion of sepsis. Transthoracic echocardiography showed moderate global hypokinesia with a left ventricular ejection fraction (LVEF) of 40-45% and a small circumferential pericardial effusion, measuring approximately 6 mm posteriorly and 5 mm anteriorly. Blood cultures grew *S. pneumoniae*. Antimicrobial susceptibility testing revealed intermediate resistance to penicillin G and amoxicillin, preserved susceptibility to ceftriaxone and levofloxacin, and resistance to doxycycline, erythromycin, and azithromycin (Table 2). 

**Table 2:** Antimicrobial susceptibility profile of Streptococcus pneumoniae isolated from blood cultures

Antibiotic	MIC/Result	Interpretation
Penicillin G	Intermediate	Reduced susceptibility
Amoxicillin	Intermediate	Reduced susceptibility
Ceftriaxone	Susceptible	Appropriate option
Levofloxacin	Susceptible	Appropriate option
Doxycycline	Resistant	Not recommended
Erythromycin	Resistant	Not recommended
Azithromycin	Resistant	Not recommended

Antibiotic therapy was escalated with oral levofloxacin (500 mg twice on day 1, then 500 mg daily). Cardioprotective therapy with bisoprolol (2.5 mg/day) and ramipril (2.5 mg/day) was initiated. Colchicine (1 mg/day) was started as adjunctive therapy for pericardial inflammation.

Extensive workup excluded alternative etiologies: the patient had no history of drug use, eosinophil count was normal, viral polymerase chain reactions (PCRs) including cytomegalovirus (CMV) blood and BioFire® multiplex panel from nasopharyngeal swab and sputum were negative, and serologies for human immunodeficiency virus (HIV), hepatitis B virus (HBV), hepatitis C virus (HCV), Parvovirus B19, and Coxsackie viruses were negative. Epstein-Barr virus (EBV) and CMV profiles indicated past infection, and autoimmune screening, including antinuclear antibody (ANA) and rheumatoid factor (RF), was negative.

Cardiac MRI performed on day 7 revealed moderate pericardial effusion, septal dyskinesia, and LVEF of 52%, without evidence of edema, hyperemia, or necrosis.

The patient defervesced by day 3 of antibiotic therapy, which was continued for 14 days. Inflammatory and cardiac biomarkers normalized, and echocardiography at three months confirmed complete recovery of cardiac function. Cardioprotective therapy was maintained for one year.

## Discussion

*S. pneumoniae* is the leading cause of CAP and is associated with the highest mortality and cardiovascular complication rate among hospitalized adults with CAP [1]. Its ability to cause pneumonia depends on evasion of mucosal defenses through neuraminidase activity and capsule shedding [3,4]. Bacteremia occurs in 20-25% of hospitalized cases [5].

In our case, *S. pneumoniae* was isolated from blood cultures. The pathogen accesses the bloodstream via lymphatic invasion or transcellular passage across epithelial and endothelial cells, the latter facilitated by pneumolysin and phosphorylcholine (ChoP), which bind the platelet-activating factor receptor (PAFr). Once in circulation, it evades phagocytosis via increased capsule expression [3].

Cardiac involvement is mediated by virulence factors ChoP and CbpA (Choline-binding protein A), which facilitate pneumococcal translocation into the myocardium [3,5,6]. Once in the myocardium, pneumococci form biofilms and create intramyocardial microlesions. Resident macrophages are destroyed via necroptosis [5], leading to contractile dysfunction and cardiomyocyte necrosis [6].

Clinically evident pneumococcal myocarditis remains rare, and the true incidence is unknown due to likely underdiagnosis; only a limited number of cases have been reported in the literature [6-12]. It typically presents with chest pain, palpitations, dyspnea, or syncope, with chest pain being the most frequent symptom. One-quarter of cases are complicated forms. ECG abnormalities (90%) and elevated troponins (≥50 %) are common in myocarditis [13]. Chest X-ray may reveal cardiomegaly or pulmonary congestion [13].

In our case, chest pain was the primary symptom suggestive of myocarditis, although the patient also presented with tachycardia and hypotension related to systemic infection. The diagnosis was supported by elevated troponins and echocardiographic abnormalities. Cardiac MRI confirmed functional impairment without inflammation or necrosis. MRI is the preferred non-invasive tool for myocarditis diagnosis and is recommended in all suspected cases [13].

Endomyocardial biopsy remains the gold standard but is reserved for selected severe or atypical presentations due to procedural risk [13]. It was not indicated in our case.

Beyond antimicrobial therapy, supportive treatment was guided by both the myocardial and pericardial components of the disease. Colchicine was prescribed in accordance with current guidelines for the management of acute pericarditis, due to concomitant pericardial involvement [14]. Its role in isolated myocarditis remains uncertain, and in our case, colchicine was used specifically for the pericardial component rather than the myocardial dysfunction.

For the left ventricular dysfunction, standard heart failure therapy with an ACE inhibitor (ramipril) and a beta-blocker (bisoprolol) was initiated. Such cardioprotective agents are recommended in patients with reduced LVEF, including those with myocarditis, to improve ventricular remodeling and long-term outcomes [15].

Standard treatment for IPD includes beta-lactams. For non-severe CAP in hospitalized, immunocompetent adults, a beta-lactam-macrolide combination or fluoroquinolone monotherapy is recommended. Severe cases require combination therapy [16]. Standard treatment for infectious myocarditis consists of targeted antibiotics against the causative organism. For pneumococcal myocarditis, no specific guidelines exist regarding adjunctive therapy or exact treatment duration, and practices vary. In our case, 14 days of antibiotic therapy were administered with a good outcome. 

Reported cases of pneumococcal myocarditis generally have favorable long-term outcomes (Table 3), though the true incidence may be underestimated due to subclinical or unrecognized presentations.

**Table 3:** Reported cases of pneumococcal myocarditis: clinical features and outcomes ITP, immune thrombocytopenic purpura; N/A, not available; Amox-clav, amoxicillin-clavulanic acid; CAP, community-acquired pneumonia; LVEF, left ventricular ejection fraction

Author	Year	Age	Sex	Comorbidities	Clinical Form	LVEF (%)	Antibiotics	Duration	Outcome
Tritar K et al.	2025	18	M	None	CAP	40-45	Amox-clav + gentamicin + levofloxacin	14 days	Favorable
Gandhi T et al. [11]	2008	42	M	None	CAP	15-20	Ceftriaxone + levofloxacin	23 days	Favorable
Ahmed AR et al. [7]	2017	52	F	None	CAP	40-45	Ceftriaxone	N/A	Favorable
Cano CB et al. [10]	2019	18	M	None	CAP	49	N/A	N/A	Favorable
Chiong YK et al. [12]	2020	17	M	None	CAP	35	Ceftriaxone	10 days	Favorable
Chang CY et al. [6]	2023	56	F	ITP, splenectomy	Meningitis	N/A	Ceftriaxone	14 days	Favorable

## Conclusions

IPD carries significant morbidity and can affect multiple organ systems, including the heart. Myocarditis may complicate pneumococcal pneumonia more frequently than currently recognized, even in immunocompetent young adults. Pulmonary and cardiac symptoms often overlap, which can delay diagnosis and appropriate treatment. Early recognition through careful clinical assessment, measurement of cardiac biomarkers, and imaging, especially cardiac MRI, can guide timely intervention and prevent long-term cardiac dysfunction. Clinicians should maintain a high index of suspicion for cardiac involvement in patients with IPD to ensure optimal management and outcomes.
